# Long-term effectiveness of the Diabetes Conversation Map Program: A prepost education intervention study among type 2 diabetic patients in Taiwan: Erratum

**DOI:** 10.1097/MD.0000000000008377

**Published:** 2017-10-20

In the article, “Long-term effectiveness of the Diabetes Conversation Map Program: A prepost education intervention study among type 2 diabetic patients in Taiwan”,^[1]^ which appeared in Volume 96, Issue 36 of *Medicine,* the number for Research Ethics Committee of Dalin Tzuchi Hospital appeared incorrectly and should be No. B10203013.
